# Obesity-Related Chronic Kidney Disease: From Diagnosis to Treatment

**DOI:** 10.3390/diagnostics15020169

**Published:** 2025-01-14

**Authors:** Elena Avgoustou, Ilektra Tzivaki, Garyfalia Diamantopoulou, Tatiana Zachariadou, Despoina Avramidou, Vasileios Dalopoulos, Alexandros Skourtis

**Affiliations:** 1 Second Department of Internal Medicine, Medical School, National and Kapodistrian University of Athens, Hippokratio General Hospital, Vasilissis Sofias 114, 11527 Athens, Greece; lina-diam@hotmail.com (G.D.); avrapeny96@gmail.com (D.A.); 2 First Department of Internal Medicine, Sismanogleio General Hospital, 37 Sismanogliou Str., 15126 Athens, Greece; ilektra.papagianni@gmail.com (I.T.); t.zachariadou.balasi@gmail.com (T.Z.); billydalo@hotmail.com (V.D.); 3 Department of Internal Medicine, Evangelismos General Hospital, 45-47 Ipsilantou Str., 10676 Athens, Greece; alex.skourtis@gmail.com

**Keywords:** obesity, chronic kidney disease, obesity-related glomerulopathy, biomarkers, RAAS blockade, ectopic lipid accumulation

## Abstract

Obesity has emerged as a global epidemic with far-reaching health complications, including its role as an independent risk factor for chronic kidney disease (CKD). Increasing evidence suggests that obesity contributes to CKD through multiple mechanisms, including chronic inflammation, hemodynamic alterations, insulin resistance, and lipid accumulation. These processes can culminate in histopathological changes collectively referred to as obesity-related glomerulopathy (ORG). This review aims to provide a comprehensive overview of the current knowledge regarding the prevalence, clinical manifestations, and pathophysiology of ORG. Furthermore, we emphasize the importance of identifying key biomarkers that facilitate the early detection of ORG. Finally, we explore emerging therapeutic strategies that offer promise in mitigating this growing global health crisis.

## 1. Introduction

Obesity and chronic kidney disease (CKD) are major public health concerns worldwide. The World Health Organization (WHO) defines obesity as “an excessive or abnormal accumulation of fat that poses a health risk” and classifies it based on the body mass index (BMI) [1]. A BMI between 25 and 29.9 kg/m^2^ is classified as overweight whereas a BMI of 30 kg/m^2^ or higher marks the threshold for obesity. While BMI is still commonly utilized, the distribution of body fat across different compartments might be a more accurate indicator of obesity. Waist circumference is frequently employed to assess visceral fat, which is particularly associated with adverse health outcomes [2,3].

Obesity is a chronic and increasingly prevalent condition, affecting approximately one in five adults in developed countries. Projections suggest that by 2030, nearly 40% of the global population could be impacted by this epidemic [1]. Obesity is more frequently present among individuals with a genetic predisposition while the degree of genetic contribution has not yet been clarified. Excess fat accumulation represents an independent risk factor for several diseases such as CKD, type 2 diabetes mellitus (T2DM), cardiovascular disease, hypercholesterole, hypertension, fatty liver disease, obstructive sleep apnea, gonadal insufficiency, osteoarthritis, gastroesophageal reflux disease, and even some types of malignancy [2,3].

CKD is characterized by the progressive decline in kidney function due to the decrease in the number of functional nephrons. KDIGO (Kidney Disease: Improving Global Outcomes)defines CKD as a glomerular filtration rate (GFR) of <60 mL/min/per 1.73 m^2^ or/and the presence of markers of kidney injury, or both, for a duration of at least three months, regardless of etiology [4]. Chronic kidney disease is divided intο stages based on the GFR and/or albuminuria. Hypertension and diabetes mellitus are recognized as leading causes of kidney failure while multiple studies have established obesity as an independent risk factor for CKD. This disease affects about 10% of the Western population and is associated with cardiovascular risk and overall mortality. The worldwide morbidity for CKD is around 12% [5] while it is also estimated to cause a 10-fold higher cardiovascular mortality than that of the general population [6]. By 2040, it is estimated that CKD will be the fifth leading cause of death, and obesity is suggested to be a major cause of these estimates [7]. The rate of kidney disease progression varies widely among individuals. Some patients progress rapidly to kidney failure while others remain stable or even improve over time. In the vast majority of patients, the disease is indolent and is usually detected in an advanced stage [6,8,9].

Given the rising prevalence of obesity and its well-established role in the development of CKD, this narrative review aims to explore the intricate relationship between obesity and kidney disease, with a focus on elucidating the underlying pathophysiological mechanisms, clinical implications, and potential therapeutic strategies to address the growing burden of obesity-related established kidney disease. Additionally, we underscore the importance of identifying key biomarkers for the early detection of obesity-related glomerulopathy (ORG) and its timely management.

## 2. Obesity-Related Glomerulopathy (ORG)

### 2.1. Overview

Several studies have demonstrated a strong association between obesity and CKD. Kidney impairment due to obesity has been defined as ORG and is increasing in prevalence [10,11].

In the United States, nearly half of the population suffers from both obesity and CKD. Several long-term cohort studies in individuals without prior kidney disease have shown that a rise in BMI is linked to the onset of albuminuria, a reduced estimated glomerular filtration rate (eGFR), and an increased risk of kidney failure. According to National Health and Nutrition Examination Survey (NHANES) data, individuals with obesity face a statistically significantly higher lifetime CKD risk compared with people with normal weight (41.0% vs. 32.5%, respectively) [12].

Interestingly, several population-based studies have reported that obesity may be a significant contributing factor in the pathogenesis of chronic kidney disease for up to 30% of patients. A meta-analysis found that each additional 1 kg/m^2^ increase in BMI was linked to a 2% higher likelihood of progressing to an eGFR below 60 mL/min/1.73 m^2^ [13]. It is still unclear whether obesity serves as a primary cause, a contributing factor, or both in the advancement of CKD [14,15,16].

Both central and general adiposity are associated with kidney disease. However, central abdominal adiposity seems to constitute an independent risk factor for kidney dysfunction. Analyses from the NHANES have revealed that abdominal obesity is independently associated with albuminuria, even in the absence of T2DM and hypertension [17]. In a study of UK biobank, a 0.06 increase in the waist-to hip ratio was associated with a 30% higher risk of CKD and a 5 kg/m^2^ higher BMI resulted in a 50% higher risk of CKD [18].

Regrettably, ORG can go undetected for years due to a lack of overt symptoms, often only becoming apparent once significant kidney dysfunction has developed. While the early detection of kidney dysfunction remains an unmet medical need, it is the key to reducing the burden of disease. Therefore, there is increasing interest in finding novel biomarkers for CKD that are easy to measure, stable, and reliable while site-specificity based on the type of injury (tubules vs. glomeruli) is also important. Currently, no single candidate biomarker fulfils these criteria effectively. Despite the development of new urinary biomarkers, many have restricted practical use in clinical settings. Combining biomarkers could enhance the accuracy of predicting ORG progression and outcomes [8,9].

### 2.2. The Pathophysiology of ORG

**Figure 1:**
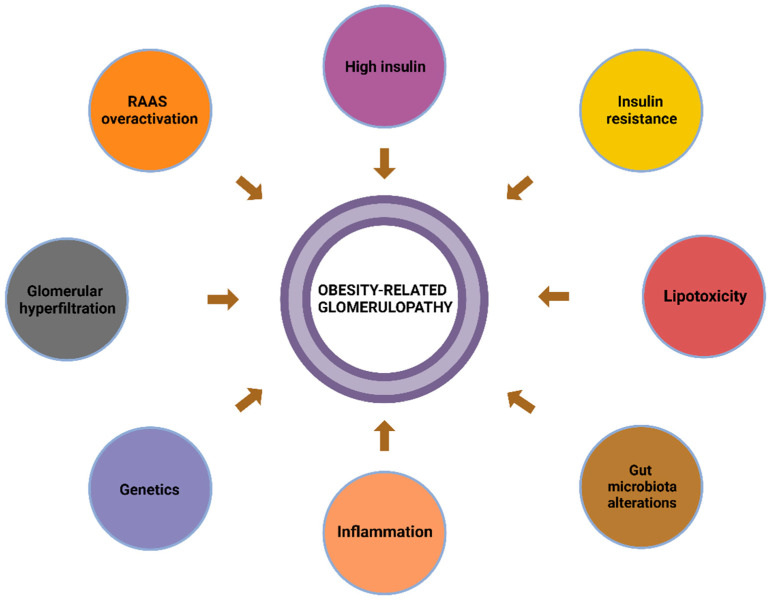
The development of obesity-related glomerulopathy (ORG) involves key mechanisms such as glomerular hyperfiltration, overactivation of the RAAS, and podocyte injury leading to fibrosis and proteinuria. Inflammatory mediators and ectopic lipid deposition further exacerbate glomerular damage through lipotoxicity and mechanical stress. Pro-inflammatory and vasoconstrictive effects of adipokines, along with hyperinsulinemia and insulin resistance, contribute to podocyte dysfunction and glomerulosclerosis. Emerging research highlights the role of microbiota alterations and genetic factors in ORG pathogenesis. Abbreviations—RAAS: Renin–Angiotensin–Aldosterone System. Created with www.BioRender.com (accessed on 12 October 2024) [20].

Whereas the role of obesity in the development of CKD is not yet fully understood, the pathophysiology of CKD has been relatively well documented. Several mechanisms contribute to the development of ORG, with the most significant being inflammation, oxidative stress, insulin resistance, glomerular hemodynamic changes, renin–angiotensin–aldosterone system (RAAS) overactivation, ectopic lipid accumulation, and changes in the gut microbiota [19] (Figure 1).

#### 2.2.1. Inflammation

While macrophages can secrete both pro-inflammatory and anti-inflammatory cytokines, in obesity, there is a predominance of inflammatory macrophages, resulting in a chronic, low-grade inflammatory state. In particular, there is a phenotypic switch from anti-inflammatory macrophages (M2) to pro-inflammatory macrophages (M1) [21]. Moreover, visceral adipose tissue secretes various cytokines with systemic effects, such as leptin, resistin, visfatin, or fetuin-A, and ROS as well, which seem to play a significant role in the pathogenesis of ORG [14,15,22].

Leptin stimulates sympathetic vascular tone and renal sodium reabsorption, leading to the hyperfiltration and overexpression of transforming growth factor β (TGF-β),which contributes to renal fibrosis and proteinuria. Resistin, in turn, increases the expression of inflammatory cytokines such as tumor necrosis factor α (TNF-α), interleukin 6 (IL-6), and interleukin 12 (IL-12). Fetuin-A has been also associated with higher levels of pro-inflammatory cytokines, decreased insulin sensitivity, and abnormal fat accumulation [23].

In sharp contrast, in obesity, there is a significant reduction in the production of adiponectin, which exhibits anti-inflammatory, cardioprotective, and insulin-sensitivity-enhancing properties. Adiponectin provides a protection for podocytes by stimulating AMP-activated protein kinase (AMPK), which in turn suppresses pathways of inflammation and fibrosis in ORG [2,14,24,25,26,27].

#### 2.2.2. Oxidative Stress

The expansion of adipose tissue also results in the accumulation of ROS, which exert detrimental effects on intracellular organelles. ROS are highly reactive molecules involved in the oxidization of lipids and proteins, which leads to cellular injury and, in particular, to glomerular and renal tubule damage associated with proteinuria. In particular, the endoplasmic reticulum’s (ER) function of maintaining cellular homeostasis and mitochondria’s crucial role in cellular metabolism are disrupted, thus inducing significant kidney dysfunction. ROS can induce ER stress leading to abnormal protein folding and apoptosis. In addition, Peroxisome-proliferator-activated receptors (PPARs) also seem to have an impact on renal function due to their key role as regulators of oxidative stress and metabolism. In studies with animal models, mutations in PPAR-γ led to inappropriate lipid accumulation resulting in kidney impairment [14,19,24,28].

#### 2.2.3. Insulin Resistance

In obese individuals, the secretion of pro-inflammatory cytokines, free fatty acids, and leptin form hypertrophic adipocytes; all contribute to the onset of insulin insensitivity, which, in turn, is associated with the dilation of the afferent arteriole and, ultimately, with high blood pressure in the glomeruli. Insulin resistance has also been associated with the onset of albuminuria. Notably, insulin is essential for normal podocyte function, morphology, and survival. It has been demonstrated that insulin resistance is associated with podocyte apoptosis and glomerulosclerosis, even in the absence of overt diabetes mellitus [14,15,28,29].

#### 2.2.4. Haemodynamic Changes

The renin–angiotensin–aldosterone system (RAAS) regulates electrolyte and fluid balance to maintain stable blood pressure. In response to low blood pressure, renin converts angiotensinogen to angiotensin I, which ACE transforms into angiotensin II. Angiotensin II acts as a potent vasoconstrictor and stimulates aldosterone release, promoting sodium reabsorption in the kidneys and fluid retention. These actions, particularly the vasoconstriction of the efferent arteriole, increase hydrostatic glomerular pressure and the eGFR [14,30].

RAAS overactivation in obesity plays a crucial role in the pathogenesis and progression of ORG. It is proposed to arise from several factors: (a) mechanical hemodynamic changes caused by the external pressure of kidney parenchyma from intra-abdominal fat and the heightened pressure within the abdominal cavity, (b) the direct production of different components of the RAAS by fat tissue, and (c) activation by the sympathetic system [14].

Notably, angiotensinogen is also produced by adipocytes. In obesity, there is an abundance of angiotensinogen in parallel with an excessive secretion of inflammatory cytokines, resulting in an aberrant activation of RAAS and systemic hypertension. Furthermore, several studies have indicated that plasma aldosterone concentrations are excessively elevated in individuals with both hypertension and obesity, especially those with central obesity. This evidence implies that people with obesity might be especially responsive to the anti-proteinuric, kidney-protective, and heart-protective effects of aldosterone blockers. Simultaneously, the sympathetic nervous system is also abnormally activated in obese individuals. This concurrent activation of RAAS and the sympathetic system reduces sodium excretion and increases its reabsorption and therefore leads to the expansion of the blood volume [14,19,24,31].

Moreover, obesity impacts the renal hemodynamics by increasing the GFR with afferent arteriole dilation, resulting in increased kidney plasma flow and consequently an increased intraglomerular pressure, eGFR, and filtration fraction. Glomerular hyperfiltration (GH) represents the central mechanism of kidney injury in ORG.GH’s pivotal role in the progression of kidney disease was first described in the case of diabetic nephropathy. Through this increased intraglomerular pressure, GH causes mechanical stress on the filtration barrier, damaging the podocytes and leading to focal segmental glomerulosclerosis (FSGS) and expanding the basement membrane causing glomerulomegaly. From a histological perspective, GH is also characterized by the expansion of glomerular and tubular structures and detachment of the glomerular basement membrane from podocytes. Clinically, GH manifests as excessive albumin excretion because of the filtration barrier injury. Albuminuria should be detected early before nephropathy is settled [2,14,19,24,25,28,32].

#### 2.2.5. Lipotoxicity

The lipid nephrotoxicity hypothesis supports the notion that renal fat accumulation in obese individuals may cause kidney injury. Moreover, perirenal adipose tissue releases free fatty acids (FFAs) and adipokines that may exacerbate intrarenal damage through lipotoxicity by increasing the production of various metabolites of FFAs. Abnormal perirenal and pararenal fat buildup also physically constricts the vessels and parenchyma of the kidney, raising renal interstitial fluid pressure and decreasing renal tubular perfusion. There have also been studies that have documented de novo lipogenesis in obesity, further promoting lipid nephrotoxicity and, consequently, the activation of inflammatory pathways [14,19].

#### 2.2.6. Alterations in the Gut Microbiota

The gut microbiota refers to the sum of bacteria, viruses, fungi, and archaea inhabiting the human intestines. These microorganisms comprise a number of 10^14^ approximately while the number of eukaryotic cells in the human body is around 10^13^. These huge numbers are suggestive of the significance of the gut microbiota in terms of the normal functioning of humans. Indeed, under normal circumstances, there is a balance between the gut microbiota and the host. However, under abnormal circumstances, the phenomenon of “gut dysbiosis” occurs, which is implicated in the development of various diseases [33]. Nowadays, obesity has been advocated to be characterized by alterations in both the diversity as well as the composition of the gut microbiota [34]. More specifically, the Firmicutes-to-Bacteroidetes ratio (F/B) has been demonstrated to be increased in most animal models, whilst in humans, the results remain controversial. Nevertheless, there is mounting evidence supporting the existence of changes in the composition and/or reductions in the diversity of the gut microbiota in obesity [34,35]. Regarding CKD in obesity, the involvement of uremic toxins is suggested to play a key role. More specifically, the levels of p-cresyl and indoxyl, which, in the liver, are converted to p-cresyl sulfate (PCS) and indoxyl sulfate (IS), are elevated in obesity-related CKD [36]. These uremic toxins are gut-microbiota-derived toxins, which, together, with trimethylamine (TMA) and branched chain fatty acids (BCFAs), are increased in cases of obesity-related CKD [24].

#### 2.2.7. Genetics

Some studies have demonstrated an upregulation of glomerular genetic polymorphisms, which is related to pro-inflammatory cytokines, fat metabolism, and insulin insensitivity. Likewise, genes linked to adiposity have been shown to be strongly connected to CKD. Zhu et al., based on data from 281,228 UK BioBank participants and by using Mendelian randomization, studied the contribution of genetic polymorphisms on obesity-related chronic kidney disease. They concluded that relationships between adiposity and CKD are largely causal [18]. However, further research in this field is anticipated, as this could potentially allow for targeted therapeutic approaches for ORG [14].

### 2.3. Histopathology of ORG

“Glomerulomegaly” is the pathological signature of the disease and consists of glomerular hypertrophy as a result of hemodynamic alterations in ORG, represented by GH. Another characteristic histopathological lesion of the disease is oligonephronia (low glomerular density). Reduced glomerular density may be strongly associated with the development of renal damage in ORG, resulting in compensatory glomerulomegaly. This adaptive glomerulopathy, in turn, results in the loss of nephron mass as a result of the prolonged detrimental effects of elevated intraglomerular pressure. FSCS may also occur alongside glomerulomegaly, affecting mainly the perihilar hypertrophic glomeruli. Interstitial fibrosis, tubular degeneration, and arteriosclerotic scars may occur before the disease manifests clinically [14].

Other characteristic features of ORG include a subtle and irregular loss of podocytes unlike the severe damage seen in primary FSGS. Additionally, immunofluorescence reveals non-specific deposits of IgM and C3, with no specific immune complexes detected. Additionally, focal diabetoid changes such as the expansion of the mesangial matrix, focal mesangial sclerosis, or the mild focal thickening of tubular basement membranes may occur even without clinical insulin resistance [37]. Moreover, lipid aggregation may be apparent in kidney biopsies of patients with obesity as intracellular lipid vacuoles in proximal tubular epithelial cells, podocytes, and mesangial cells [38].

### 2.4. Clinical Presentation

A gradual rise in subnephrotic proteinuria (less than 3.5 g/d) with normal urinary sediment and without hypoalbuminemia is the most frequent clinical presentation of ORG, occurring in an overweight individual and, in the absence of additional factors, leading to renal damage. Even without treatment, the proteinuria in ORG progresses slowly. Ultimately, as many as 30% of individuals with ORG may develop nephrotic syndrome with protein excretion exceeding 3.5 g per day but without hypoalbuminemia or other features of the syndrome. There is, however, an elevated risk of advancement to kidney failure. The clinical presentation observed once CKD is established reflects the typical manifestations of kidney damage [14,31,39].

## 3. Biomarkers—Overview

As already mentioned, CKD is an indolent disease in the early stages with no obvious clinical symptoms until severe kidney damage has occurred. The absence of sensitive and specific biomarkers complicates and delays early diagnosis. The accurate assessment of renal function in obese patients is essential for staging kidney disease, monitoring its progression, and adjusting medication dosages. While the non-invasive evaluation of ORG is now based on the albuminuria, emerging biomarkers along with advanced techniques in proteomics and metabolomics could enable a more prompt diagnosis of ORG. In addition, kidney imaging, through the evaluation of ectopic kidney fat, could also facilitate the detection and the monitoring of the progression of ORG [6,9,40].

### 3.1. Serum Creatinine (SCr)

Creatinine is a breakdown product of creatine phosphate in skeletal muscles. It is derived from the muscle metabolism and consumption of meat or creatine supplements as well. Creatinine is eliminated from the bloodstream by the kidneys, primarily through glomerular filtration and to a lesser extent by active secretion in the proximal tubules. There is minimal to no tubular reabsorption of creatinine. Due to the fact that its concentration is affected by multiple parameters, such as muscle mass, diet, body weight, and hydration status, SCr is a less reliable diagnostic biomarker in obese individuals; οbesity is linked to increased muscle mass and creatinine production, which may lead to an apparently lower estimated glomerular filtration rate (eGFR) in individuals with obesity [41]. Moreover, serum creatinine is not sensitive to small variations in kidney function. Kidney damage has usually occurred resulting in CKD before SCr becomes abnormal [25,42,43].

### 3.2. CystatinC

CystatinC is a low-molecular-weight (13.3 kDa) protein produced at a constant rate by all nucleated cells. It acts as a cysteine proteinase inhibitor and is freely filtered by the renal glomeruli. Unlike serum creatinine, cystatinC levels are minimally influenced by factors such as age, gender, ethnicity, nutritional status, or muscle mass, making it a more reliable marker for kidney function across diverse patient populations. However, epidemiological studies indicate that it is associated with the body mass index, likely due to a direct relationship between cystatinC and fat mass [44]. CystatinC is more sensitive than creatinine for detecting early and subtle changes in renal function. However, its widespread use for chronic kidney disease screening is debated due to its significantly higher cost. Despite this, it is particularly beneficial for patients with abnormal creatinine excretion, such as those with sickle cell disease, congenital heart disease, or low muscle mass or those undergoing chemotherapy. CystatinC is not only a reliable diagnostic marker for renal impairment but also an independent predictor of morbidity and mortality. Studies have linked elevated serum cystatinC levels to a higher risk of progression to kidney failure in patients with type 2 diabetes, kidney injuries, and liver diseases. Additionally, it is a strong and independent predictor of cardiovascular risk in patients with CKD [42,45,46].

### 3.3. Glomerular Filtration Rate (GFR)

The glomerular filtration rate measures the volume of fluid filtered by the kidney’s glomeruli into the Bowman’s capsule per unit time. It equals the renal clearance rate for solutes that are freely filtered and neither reabsorbed nor secreted by the kidneys. Various equations have been developed to estimate the GFR (eGFR), typically using serum creatinine or cystatin C levels. The two more widely used equations are the Modification of Diet in Renal Disease (MDRD) and the Chronic Kidney Disease Epidemiology Collaboration (CKD-EPI) equations. Each of them has its own limitations and can be time-consuming and burdensome for patients, but can generally be used with a reasonable level of reliability in non-obese individuals. The MDRD Study introduced the original equation in 1999 to account for standardized creatinine assays. This equation quickly became one of the most widely used methods for estimating the glomerular filtration rate in clinical practice. However, in 2009, the CKD-EPI equation was developed to improve upon the MDRD formula, offering more accurate estimates for individuals with higher eGFRs (>60 mL/min/1.73 m^2^). Additionally, the CKD-EPI also developed equations that incorporate both serum creatinine and cystatin C, improving the precision of eGFR estimates [47,48]. It has been noted that the CKD-EPI equation provides an accurate estimation of GFR for estimated glomerular filtration rates lower than 60 mL/min/1.73 m^2^ as long as the BMI is below 40 kg/m^2^. For higher BMIs, however, the assessment of GFR by formulas is debatable as it often does not accurately represent actual kidney function [25,49].

Lopes Martinez et al. conducted a study on over 900 overweight or obese individuals, with or without CKD, to evaluate kidney function using 56 creatinine- or cystatin-C-based equations. They found significant errors in eGFR estimates compared to measured GFR (mGFR) using plasma iohexol clearance, particularly in equations incorporating weight or height. Adjusting the mGFR/eGFR for body surface area (BSA) frequently underestimated kidney function by at least 10 mL/min in 25% of cases. The study suggested discontinuing the practice of BSA adjustment [50]. Alaje et al. evaluated kidney function among 59 obese subjects via creatinine-and cystatin-based equations. They showed that there was a significant difference between the eGFR cystatin C (75.4 ± 38.9 mL/min/1.73 m) and the eGFRCr (97.4 ± 21.4 mL/min/1.73 m). These findings align with earlier research indicating that cystatin C is a more effective marker of kidney impairment in overweight individuals compared to creatinine [25]. Lemoine et al., in their study that included 209 CKD obese patients in stages 1–5, showed that the GFR equations are not as accurate when kidney function is preserved or in the case of hyperfiltration [51].

Some researchers recommend utilizing a gold standard method (such as inulin or iohexol plasma clearance) for assessing kidney function in obese individuals [51,52]. However, these techniques are not commonly accessible, making them impractical for routine clinical use. Measuring the glomerular filtration rate through the transdermal clearance of fluorescent tracers could provide a feasible alternative for these patients although further research seems mandatory [53,54].

### 3.4. NGAL (Neutrophil-Gelatinase-Associated Lipocalin)

NGAL is an iron-transporting protein of the lipocalin family, primarily secreted by activated neutrophils during bacterial infections but also found in various tissues including renal tubular epithelial cells. It is filtered by the glomeruli and reabsorbed in the proximal tubules. NGAL production increases in response to kidney injuries such as ischemic, nephrotoxic, or septic damage. It appears in plasma within two hours of acute kidney injury (AKI), peaking at six hours and remaining elevated for about five days before declining. This early detection makes NGAL a valuable biomarker for AKI, preceding noticeable declines in the eGFR. AKI also leads to an increase in urine NGAL levels, with both serum and urine NGAL rising 24 h earlier than creatinine levels. Nowadays, it has been suggested that NGAL is a sensitive biomarker of tubular damage and not of kidney dysfunction. Multiple studies have proven that NGAL is a sensitive diagnostic and predictive biomarker of chronic kidney disease as well [55,56]. In patients with T2DΜ, urinary NGAL levels rise before the appearance of traditional markers (albuminuria, elevated Scr) indicating subclinical tubular damage. Hence, urinary NGAL could serve as an early biomarker for the early diagnosis of diabetic nephropathy [55,56,57,58,59,60].

Kapoula et al. conducted a meta-analysis of 22 studies assessing NGAL as a diagnostic marker for diabetic nephropathy. They found that both serum and urinary NGAL levels increased with the progression of albuminuria and declining GFR, with the highest NGAL concentrations observed in patients with the most severe disease [55].

### 3.5. KIM-1 (Kidney Injury Molecule-1)

KIM-1 is a transmembrane glycoprotein involved in immune response activation during viral infections. While undetectable in healthy kidneys, KIM-1 levels rise within 24 h of acute kidney injury, making it a key biomarker for AKI. It is also elevated in chronic kidney disease but is expressed only in actively damaged renal tubules, with no expression in atrophic or fibrotic regions. As fibrosis progresses, KIM-1 expression decreases [61]. Most studies indicate that KIM-1 levels are elevated in T2DM and are associated with an increased urine albumin-to-creatinine ratio [59,62]. However, in a follow-up study involving 527 adults with T1D, Bjornstad et al. (2018) found that the urine albumin-to-creatinine ratio and reductions in the GFR were linked only to NGAL and not to KIM-1 [63].

### 3.6. Gal-3 (Galectin-3)

Gal-3 is a galactoside-binding lectin mainly expressed in the proximal tubules and collecting ducts of the kidney. It is essential in kidney fibrogenesis, contributing to CKD. It is abundantly secreted by renal macrophages in all inflammatory kidney disorders [64].

#### NGAL, KIM-1, and Gal-3 in ORG

The overproduction of these molecules in obese individuals has been primarily documented in the pediatric population. Şen et al. have demonstrated that NGAL levels are significantly higher in pediatric non-diabetic, obese, or overweight patients with insulin resistance (IR) than those without these conditions. They did not find a difference in the levels of urinary KIM-1 or serum cystatin between the IR and the control group. The findings indicate that NGAL measurements could serve as an early biomarker for kidney injury in obese patients with insulin resistance, even in the absence of diabetes and microalbuminuria, and that these patients are at risk of early kidney damage [65]. Mackowiak-Lewandowicz K et al. recently studied 142 obese adolescents divided into ‘elevated GFR’, ‘normal GFR’, and ‘decreased GFR’ groups aiming at determining potential predictors of ORG. They found that most obese study participants with “normal GFR” who later developed CKD had initially higher urine NGAL concentrations compared to obese adolescents with “normal GFR” who did not develop kidney disease. Therefore, they suggested NGAL as a useful predictor of CKD development in individuals with “normal GFR”. All study participants exhibited albuminuria, as well as increased urine Gal-3 concentrations, regardless of their GFR levels, implying its high efficiency in detecting ORG at an early stage [64].

In another study that compared obese and normal-weight prepubertal children, both of whom had no overt kidney impairment based on the GFR, and normal SCr values and urinary NGAL and KIM-1 and serum cystatin values were significantly higher in obese individuals. A strong positive correlation was found between all these three biomarkers and indices of adiposity (BMI, waist-to hip-ratio). Obese patients had also significantly larger kidneys on ultrasound, confirming that organ hypertrophy occurs early in ORG [66]. In sharp contrast, a previous study involving obese children had reported no association between urinary KIM-1 levels and kidney injury [67].

### 3.7. Klotho

Klotho is a transmembrane protein predominantly found in the kidneys (in proximal and distal tubular cells), but also in the brain and in endothelial cells. It acts as an aging suppressor. In humans, elevated Klotho levels are linked to lower risks of CKD, CVD, and mortality while Klotho deficiency has been correlated with impaired kidney function. As its levels correlate with the degree of kidney disease, Klotho has been described as a sensitive and early biomarker of CKD. A recent extensive cohort of U.S. adults, demonstrated an inverse relationship between serum Klotho levels and the incidence of metabolic syndrome and proposed recombinant Klotho protein administration as a promising new treatment strategy with already favorable outcomes in mouse models [40,68,69,70].

### 3.8. The Role of MicroRNAs in ORG and CKD-Overview

miRNAs are small, noncoding RNAs that regulate gene expression by either promoting mRNA degradation or inhibiting translation. A single miRNA can target and regulate multiple genes. Mitchell et al. first demonstrated that miRNAs are present in human plasma and remain stable despite the high RNAse activity in plasma. This stability is due to their encapsulation in exosomes, which protect them from circulating ribonucleases [71]. MicroRNA expression profiles in body fluids, such as blood or urine, could be used as clinical biomarkers for the early detection of kidney fibrosis and established kidney disease. miRNAs seem to fulfil the specificity, sensitivity, and reproducibility criteria needed for reliable, non-invasive biomarkers as long as accurate normalization strategies and proper controls are applied for precise quantification. Since miRNAs can regulate molecular pathways and their expression may be disease-specific, they appear to offer diagnostic potential. For example, serum miR-148b is specific for IgA nephropathy. However, it is noteworthy that a single miRNA may be implicated in multiple different diseases [72].

#### 3.8.1. miRNAs and Kidney Homeostasis

miRNAs contribute to the progression of CKD by regulating mRNAs that are involved in maintaining renal homeostasis. Some miRNAs have been shown to be tissue-specific, with particular expression in the kidney. For instance, the concentrations of miR-192, miR-194, miR-204, miR-215, and miR-216 are higher in the kidneys than in other organs. Conversely, low or absent levels of certain miRNAs in the kidney may promote the expression of proteins essential for kidney function. The microarray profiling of kidney biopsy samples from CKD patients showed that 40 miRNAs were upregulated while 76 miRNAs were downregulated [73,74].

#### 3.8.2. miRNAs as Predictive Markers: Diagnostic and Predictive Potential

miRNAs can be used not only as diagnostic but as predictive markers as well. Studies indicate that miRNA levels are generally reduced as CKD progresses, though the precise mechanism behind this decrease remains unclear. An analysis of miRNA expression in kidney biopsies showed that miR-223 levels were higher in patients with progressive kidney disease compared to those with stable kidney disease, suggesting that this miRNA may worsen renal dysfunction [75]. Additionally, targeting the silencing of miRNAs associated with albuminuria or restoring miRNA function in kidney diseases where miRNAs are downregulated could be an important therapeutic strategy. Preliminary results from animal studies on the use of anti-miRNAs are promising [76,77].

#### 3.8.3. Significance of miRNAs in ORG

In summary, miRNAs show great potential as reliable biomarkers for kidney diseases, including ORG, due to their tissue specificity and stability in various biological samples. Several miRNA biomarkers have been identified in kidney disease, offering opportunities to improve diagnostic accuracy, monitor disease progression, and evaluate treatment responses [76].

#### 3.8.4. The Impact of Lifestyle on miRNA Expression

MicroRNA expression can be influenced by both diet and physical activity, which also impact obesity. Diet affects protein synthesis and microRNA processing, with dietary changes linked to epigenetic modifications that alter cellular signaling. Adjusting diet may improve microRNAs related to kidney diseases, even without significant weight loss. Specific microRNA families, such as miR-17, miR-21, and miR-200, are affected by diet, with high-fat diets notably downregulating miR-200b and miR-200c [78]. Likewise, physical activity also impacts microRNA expression, independent of weight loss, and a meta-analysis showed that it influences miRNAs like miR-21, miR-126, miR-192, miR-193b, and miR-221 in diabetic and prediabetic patients. Circulating microRNA profiles could, therefore, be useful for monitoring responses to various therapeutic interventions [8,79].

#### 3.8.5. Urinary miRNAs as Biomarkers: Challenges and Opportunities

Urinary microRNAs have become valid alternatives to tissue and circulating serum microRNAs on account of the identification of kidney-specific microRNAs and reliable methods for processing them within urinary exosomes. However, isolating urinary exosomes is particularly challenging due to the complex composition of urine, which contains various substances that can contaminate exosome samples. Moreover, ultracentrifugation, the gold standard for exosome isolation from biological fluids, including urine, may not always be accessible in some laboratories [8].

Table 1 presents the associations identified between various miRNAs and different kidney diseases.

### 3.9. The Role of Imaging Modalities

Kidney ultrasonography is a non-invasive, widely available, and low-cost imaging test that can track structural changes and kidney fibrosis in ORG over time. Color Doppler ultrasonography enables the evaluation of intrarenal blood flow and hemodynamics. Contrast-enhanced ultrasonography offers the potential for assessing kidney perfusion in the early stages of nephropathy. The thickness of pararenal and perirenal ultrasonographic fat (PUFT) may serve as a valuable tool to show the ectopic lipid accumulation in the kidney (fatty kidney) [88]. Moreover, ultrasound elastography may reveal the presence and degree of kidney fibrosis. Therefore, for assessing obesity-related CKD, renal ultrasonography, due to the multi-faceted features that may be described together with its low-cost and non-invasive character, remains the most widely used imaging modality [89]. Due to the increased difficulty of interpreting ultrasonography in patients with obesity, Tc-99m dimercaptosuccinic acid (Tc-99m-DMSA) scintigraphy may be considered more readily in these cases [90].

Computed tomography offers an accurate and non-invasive assessment of kidney structure and function, including of the ectopic fat in ORG by measuring adipose tissue density in Hounsfield Units. Renal sinus fat (RSF) is independently associated with reduced kidney function regardless of intra-abdominal fat levels.

MRI is also a valuable imaging method for measuring both intrarenal and perirenal lipid accumulation in the kidney that offers high-resolution images. Therefore, MRI contributes to the early identification of kidney impairment in ORG [14,24].

## 4. Therapeutic Approaches

### 4.1. Lifestyle Recommendations

Given that ORG is due to excess body fat, lifestyle recommendations to reduce body weight in obese individuals with or at risk for CKD appear justified and, in fact, weight loss has emerged to be a prerequisite in the fight against obesity. However, the exact amount of weight loss required to potentially reduce, or at least postpone, kidney damage is still largely unknown. Non-pharmacological treatment strategies play a central role in managing CKD and obesity. A healthy and balanced diet and physical exercise are the first steps to counter obesity-related disease. There is accumulating evidence suggesting that a significant reduction in proteinuria and improvement in kidney function are associated with decreases in the BMI [91,92,93,94,95]. With the advent of new incretin-based therapies, lifestyle modifications are increasingly seen as complementary to pharmacological treatments and bariatric surgery, rather than as the initial approach. However, they should complement rather than replace lifestyle modifications. Lifestyle intervention remains the cornerstone of obesity care, forming the foundation for effective treatment [96].

### 4.2. Bariatric Surgery

Clerte et al. studied the impact on mGFR in 16 obese individuals ([BMI]: 43.9 ± 7.3 kg/m^2^) six months after bariatric surgery. They concluded that renal function showed a statistically significant improvement after surgery, along with a statistically significant reduction in the BMI (BMI after surgery 35.2 ± 5.7 kg/m^2^). In addition, in patients with GH, the GFR had been normalized at the follow-up, suggesting that glomerular dysfunction can be reversed in some individuals. They also demonstrated the superiority of the mGFR using plasma iohexol clearance in comparison to the MDRD and CKD-EPI equations when it comes to obese individuals. The latter two equations seem to underestimate the GFR in obese patients and do not detect hyperfiltration in most cases [2]. Other studies have also demonstrated the positive impact of bariatric surgery on reducing the BMI and simultaneously improving kidney function [97,98].

Bariatric surgery has a more significant positive impact on renal function compared to lifestyle modifications alone, likely due to the greater and more sustained weight loss it achieves. As a result, substantial weight loss from bariatric surgery may be seen as an appealing treatment option for patients with severe obesity and CKD. However, the risk of postoperative adverse effects, such as deep vein thrombosis, pulmonary embolism, and infections, should be taken into consideration [14]. Overall, bariatric surgery poses a slightly higher risk for patients with CKD compared to those without it, but the benefits of weight loss likely outweigh this increased risk [99].

### 4.3. Pharmaceutical Interventions

#### 4.3.1. RAAS Blockade

Given the central role of RAAS in the pathophysiology of ORG, pharmacological RAAS blockade is a reasonable therapeutic approach. The available treatment options include angiotensin-converting enzyme inhibitors (ACEIs) and angiotensin II type I receptor blockers (ARBs), both of which protect kidney function by reducing hyperfiltration and proteinuria. According to KDIGO guidelines, RAAS blockers are recommended for all patients with CKD and albuminuria due to their proven benefits in slowing disease progression and reducing cardiovascular risk [100]. However, their clinical effectiveness in patients with obesity but without proteinuria, who are at high risk of developing CKD, remains uncertain [101]. This raises questions about whether the therapeutic benefits of RAAS inhibitors extend to this subgroup as the absence of proteinuria might indicate differing pathophysiological mechanisms or treatment responses in these patients.

Mineralocorticoid receptor antagonists (MRAs), including steroidal types like spironolactone and eplerenone, as well as non-steroidal options (nsMRAs) like finerenone, effectively preserve kidney function in obese patients with proteinuric nephropathies. They achieve this by significantly and sustainably reducing proteinuria [14].

Morales et al. demonstrated that adding the mineralocorticoid-receptor antagonist (MRA) spironolactone may provide additional renal benefits for patients with obesity and proteinuria who are already being treated with RAAS blockers [102]. Non-steroidal drugs, such as finerenone, offer promising therapeutic options for improving cardiorenal outcomes in individuals with CKD and diabetes who are already receiving optimized treatment with an ACE inhibitor or ARB. It is noteworthy that hyperkalemia occurs less often with nsMRAs than with steroidal options. In patients with CKD and T2DM, treatment with nsMRAs has been associated with a reduction in critical heart-related outcomes and diabetic nephropathy advancement, although without weight loss. However, further research is needed to clarify the impact of finerenone on non-diabetic individuals with chronic kidney disease [103].

#### 4.3.2. Sodium–Glucose Cotransporter-2 Inhibitors (SGLT-2)

SGLT-2 inhibitors, including empagliflozin, canagliflozin, and dapagliflozin, are relatively new antidiabetic agents that provenly reduce major cardiovascular complications, hospital admission in heart failure patients, and the advancement of CKD in patients with T2DM and obesity. In the clinical trial CREDENCE, the adverse kidney events (doubling of serum creatinine, kidney failure, and death from renal or cardiovascular causes) in patients with advanced diabetic kidney disease (DKD) on canagliflozin were reduced by more than 30% [104]. The EMPA-REG trial showed that empagliflozin significantly benefits kidney function in patients with type 2 diabetes at high cardiovascular risk, reducing the progression of nephropathy, doubling of serum creatinine, macroalbuminuria, and the need for kidney replacement therapy compared to placebo [105].

The large-scale DAPA-CKD trial, with over 4000 patients with chronic kidney disease, both with and without diabetes, demonstrated significant improvements in kidney function among those treated with dapagliflozin, regardless of diabetes status. The primary composite endpoint included a greater-than-50% sustained decline in eGFRs, the onset of kidney failure, and cardiovascular or kidney death [106].

SGLT-2 inhibitors benefit renal function through multiple mechanisms, including reducing sodium and glucose reabsorption in the kidneys. This increases sodium delivery to the macula densa, promoting afferent arteriole vasoconstriction and alleviating GH. As a result, they promote glycosuria, reduce albuminuria, and slow eGFR decline. However, while SGLT-2 inhibitors provide a stable antidiabetic effect by lowering blood glucose levels, their effect on natriuresis is transient and modest. SGLT-2 inhibitors can promote weight loss in individuals with T2DM viaglycosuria, which results in a caloric deficit. However, the weight reduction due to SGLT-2 inhibitors is usually minimal to modest (initial weight loss of 1–3 kg during the first week of treatment, gradually slowed over the following months and then stabilized) [107]. Accordingly, SGLT-2 inhibitors provide kidney protection in both obese and non-obese individuals by slowing the decline in the glomerular filtration rate, reducing albuminuria, and delaying kidney failure. These benefits are independent of weight loss [14,103,108].

#### 4.3.3. Glucagon-like Peptide-1 Receptor Agonists (GLP-1 RAs)

Glucagon-like peptide-1 receptor agonists (GLP-1 RAs) stimulate insulin secretion, decrease glucagon release from the pancreas, improve insulin sensitivity, and increase feelings of satiety by targeting receptors in the brain, resulting in sustained weight reduction in individuals both with and without T2DM. Therefore, these drugs seem very promising for the management of ORG. Apart from their use as antidiabetic medications in patients with type 2 diabetes, they have been approved for weight management in individuals who are overweight or obese [109,110]. Semaglutide and dulaglutide also lower the cardiovascular risk in people with T2DM. Substantial decreases in glycated hemoglobin and body weight have been demonstrated with semaglutide and the first dual GLP-1/GIP agonist, tirzepatide. Tirzepatide combines the advantageous effects of both incretins into one co-agonist. Indeed, the SURPASS-2 clinical trial demonstrated a more pronounced weight loss in patients on tirzepatide than on semaglutide [111] and a statistically significant improvement in glycemic control when added on standard medication with insulin (SURPASS-5) [112].

Ongoing clinical trials are examining the effectiveness of a combination drug that includes semaglutide and the extended-release amylin analogue cagrilintide (CagriSema), as well as retatrutide, a triagonist targeting GLP-1, GIP, and glucagon, for weight reduction and the treatment of ORG. In summary, considering their multi-targeted efficacy against hyperglycemia, hyperinsulinemia, and excess fat tissue, GLP-1-based therapies may serve as valuable therapeutic options for tackling ORG.

While studies on GLP-1 RAs focusing on primary kidney outcomes have not been released, secondary endpoints from cardiovascular trials have shown encouraging results, indicating a possible kidney-protective effect. In the STEP 2 clinical trial, overweight or obese subjects with T2DM demonstrated on semaglutide a pronounced and dose-dependent improvement in albuminuria, measured as the UACR at week 68 (reduction by 32.9% with 2.4 mg semaglutide vs. placebo). The impact of semaglutide on the UACR was more significant in participants with macro- or microalbuminuria than in those with normoalbuminuria, reinforcing earlier findings regarding the anti-proteinuric effect of semaglutide [113].

Additionally, GLP-1 RAs lower blood pressure by inhibiting the RAAS and promoting natriuresis through proximal tubule Na/H exchanger 3 blockage. They also protect the kidneys by reducing inflammation and fibrosis, key factors in chronic kidney disease progression [14,24,103].

#### 4.3.4. Other Agents

Metformin, apart from lowering plasma glucose, has reno-protective effects by delaying podocyte apoptosis, inhibiting inflammatory cytokine secretion, and diminishing fat deposition [114]. In contrast, in a retrospective study, ongoing metformin therapy for at least 6 months was found to result in a greater decline in the glomerular filtration rate in patients with diabetes and moderate CKD, mainly through metformin-associated lactic acidosis [115], and other studies have described metformin use as an independent risk factor for mortality [116].

Melatonin is a hormone produced by the pineal gland. It is primarily secreted at night and is vital for regulating circadian rhythms and, ultimately, the biological clock. Melatonin also plays a crucial role in anti-inflammatory, antioxidant, and cell-protective pathways through its receptors widespread throughout the human body. In the kidney, melatonin exhibits mainly anti-inflammatory and anti-oxidative effects, acting as an efficient ROS scavenger. Moreover, melatonin’s anti-obesity properties have been predominantly related to its inhibition effects on the inflammasome. In particular, the NLRP3 inflammasome is involved in obesity-related kidney disease development. Experimental data advocate melatonin as a promising reno-protective agent, which could improve and even delay the progression of kidney disease in persons with obesity [117,118,119].

Table 2 depicts major studies regarding therapeutic approaches in obesity-related CKD and their findings.

### 4.4. Future Perspectives and Challenges

The fundamental mechanisms behind the development of ORG are intricate and not yet completely understood. However, there is accumulating evidence supporting that kidney impairment associated with obesity is independent of the classic risk factors for CKD such as hypertension and T2DM [128,129].

Table 3 illustrates the main differences between obesity-related CKD and other causes of CKD.

An urgent need for innovative and targeted approaches towards understanding, diagnosing, and managing ORG more deeply has emerged, an ambition still impeded by knowledge gaps that should be bridged. The development of new urinary and blood-based circulating biomarkers, adjusted to body surface area, for the diagnosis of ORG in the early stages is of paramount importance. Apart from diagnostic biomarkers, prognostic and predictive ones that would enable the stratification for disease progression and, ultimately, the identification of responders to treatment remains the main problem. Similarly, novel kidney imaging or genetic markers could facilitate the identification and following up of high-risk patients. Further research and longitudinal studies are needed to shed light upon diagnosing obesity-related glomerulopathy.

## 5. Conclusions

From a therapeutic scope, several emerging therapies have already shown promising, preliminary results for ORG. Therapeutic strategies with anti-proteinuric effects, including weight loss and RAAS inhibition, postpone the progression to kidney failure and are the current first-choice interventions for ORG. Antidiabetic agents such as SGLT-2 and GLP-1 RAs seem to be very promising. Large-scale trials with SGLT-2 conducted in subjects with and without T2DM have proved their nephroprotective role. Dual GLP-1/GIP and triple G receptor agonists are relatively novel drugs that have emerged as potential therapeutic strategies for this condition. However, only future large clinical trials will be able to evaluate whether all these drugs are effective in individuals with obesity-related glomerulopathy. In summary, studies specifically focusing on obese individuals with CKD are essential. Every aspect, from diagnostic techniques to therapeutic interventions, deserves careful consideration to enhance our understanding and ultimately succeed in addressing ORG.

## Figures and Tables

**Table 1:** Associations between microRNAs and various kidney disorders identified in multiple studies. Abbreviations—CKD: Chronic Kidney Disease; IgA: Immunoglobulin A; miR: MicroRNA.

Kidney Disorder	MiRNA
IgA nephropathy	miR-148b, miR-let-7b [80]
Kidney fibrosis	miR-200, miR-433, miR-21 [81,82,83]
Hypertensive nephrosclerosis	miR-200a/b, miR-141, miR-429, miR-192 [84]
Progression of CKD	miR-223-3p, miR-93-5p [85]
Diabetic nephropathy	miR-126, miR-770 [86]
Obesity-Related Glomerulopathy	miR-21, miR-29, miR-200, miR-146 [8,87]

**Table 2:** Summary of major studies on therapeutic approaches in obesity-related chronic kidney disease and their key findings. Abbreviations—ACEI: Angiotensin-Converting Enzyme-Inhibitors; ARΒs: Angiotensin Receptor Blockers; BMI: Body Mass Index; BSA: Body Surface Area; CKD: Chronic Kidney Disease; DM: Diabetes Mellitus; DDP-4i: Dipeptidyl peptidase 4 inhibitors; eGFR: Estimated Glomerular Filtration Rate; EXE: exenatide; GLP-1α agonists: Glucagon-like-peptide-1 agonists; MRA: Mineralocorticoid-Receptor Antagonist; ORG: Obesity-Related Glomerulopathy; RAS b: Renin Angiotensin System blocker; T2DM: Type 2 Diabetes Mellitus; SGLT2i: Sodium–Glucose Transport Protein 2 inhibitor; UACR: Urinary Albumin Concentration Ratio; WC: Waist Circumference; WHR: Waist-to-Hip-Ratio.

Title of the study	Study Characteristics	Outcome of the Study	Reference
Beneficial effects of weight loss in overweight patients with chronic proteinuric nephropathies	BMI > 27 kg/m^2^ With or without T2DM Not on anti-proteinuric drugs - 30 participants - 2:1 (low-calorie normoproteinic diet: usual dietary intake for 5 months)	- Moderate weight loss in overweight patients with chronic proteinuric nephropathies induces a significant decrease in proteinuria	Morales et al. [92]
The reno-protective effects of weight loss on patients with ORG	- Observational prospective study with 63 patients BMI ≥ 28 kg/m^2^ Kidney biopsy—proven ORG - Intervention: hypocaloric diet and aerobic exercise - Three groups based on weight change from baseline (>3% decrease in BMI, stable weight, >3% increase in BMI)	- The proteinuria was reduced by 51.3% in the decreased BMI group and increased by 28.78% in the increased BMI group. No difference was noted in kidney function among these groups	Shen et al. [93]
Effect of an Intensive Weight-Loss Lifestyle Intervention on Kidney Function: A Randomized Controlled Trial	- Randomized clinical trial - 6719 participants: Age 55–75 years BMI 27–40 kg/m^2^ - Intervention group: Mediterranean - Diet and lifestyle - Intervention for 1 year - Control group: usual care advice for 1 year	- Mean 1-year weight loss: 3.7 kg in the intervention group and 0.7 kg in the control group - After 1 year, eGFR declined by 0.66 and 1.25 mL/min/1.73 m^2^ in the intervention and control groups, respectively	Diaz-Lopez et al. [94]
Very Low-Calorie Ketogenic Diet: ASafe and Effective Tool for Weight Loss in Patients With Obesity and Mild Kidney Failure	- Observational prospective study - 92 overweight patients with eGFR ≥ 60 mL/min/1.73 m^2^ - Intervention: net carbohydrate intake 20–50 g/day, protein intake 1–1.4 g/kg of ideal body weight/day, and lipid intake 15–30 g/day	- Significant decrease inBMI;improvement inblood pressureandglucose and lipid metabolism - 27.7% of patients with mild CKD reported an improvement inkidney function	Bruci et al. [95]
Renal Resistive Index Predicts Post–Bariatric Surgery Renal Outcome in Nondiabetic Individuals with Severe Obesity	- Observational prospective study - 25 participants who underwent Roux-en-Y gastric bypass Morbid obesity No DM Age < 60 years No eGFR < 60 mL/min/1.73 m^2^ and/or micro- or macroalbuminuria	- Serum creatinine and eGFR showed no change after intervention while mGFR upon adjustment for BSA showed a significant increase in 1 year - Mean BMI and waist circumference reduction achieved nearly 30% in 1 year	Solini et al. [97]
Effect of Bariatric Surgery on CKD Risk	2144 adults who underwent bariatric surgery March 2006–April 2009	- Reduction inCKD risk up to 78% by year 1 after bariatric surgery	Friedman et al. [98]
Semaglutide and Albuminuria Reduction Trial in Obese Individuals Without Diabetes (SMART)	- 24-week randomized double-blind phase 3 study - Treatment with either semaglutide s.c. 2.4 mg once weekly or placebo Adults BMI ≥ 27 kg/m^2^ Albuminuria 30 mg/g–≤3500 mg/g eGFR ≥ 25 mL/min/1.73 m^2^ No DM	- Completed - Awaiting results	NCT04889183 Last update: 5 June 2024
Pharmacokinetics, Safety and Tolerability of Oral Semaglutide in Subjects with Renal Impairment	- Multicentre phase 1 study - Treatment with 5 mg p.o semaglutide for 5 days followed by 10 mg for 5 days 18–85 years BMI 18.5–40.0 kg/m^2^ No peritoneal dialysis or kidney transplantation - 71 patients	- The pharmacokinetics of oral semaglutide up to 10 mg are not affected by kidney impairment, even kidney failure - Dose adjustment for oral semaglutide is not required for patients with kidney dysfunction	Granhall et al. [120]
Once-Weekly Semaglutide in Adults with Overweight or Obesity (Research Study Investigating How Well Semaglutide Works in People Suffering From Overweight or Obesity: STEP-1)	- Double-blind trial - 1961 participants BMI > 30 kg/m^2^ No DM - Intervention: once-weekly 2.4 mg semaglutide sc, or placebo, plus lifestyle intervention	- Sustained body weight reduction to week 68 of up to 15% in the semaglutide group as compared with 2.4% with placebo	Wilding et al. [121]
The Effect of Retatrutide Once Weekly on Cardiovascular Outcomes and Renal Function in Adults Living With Obesity (TRIUMPH-OUTCOMES)	Interventional clinical trial, phase 3 Study >45 years BMI ≥ 27 kg/m^2^ With or without T2DM (Hb1Ac < 10%) Established atherosclerotic cardiovascular disease - Intervention: sc retatrutide or placebo	- Still recruiting (Last update: 27 September 2024)	NCT06383390
Effects of exenatide on urinary albumin in overweight/obese patients with T2DM: a randomized clinical trial	Randomized controlled trial 159 Participants 18–65 years HbA1c ≥ 6.5%; BMI ≥ 24 kg/m^2^ Not on RAS blockers Intervention: sc exenatide (GLP-1α) 5 μg b.i.d. for 4 weeks and 10 μg b.i.d. for 20 weeks or insulin glargine	- EXE significantly reduces the UAC at week 12 and 24 in contrast to insulin glargine - Decrease on BMI by 2.005 kg/m^2^ on EXE group and by 0.060 kg/m^2^ on insulin group (wk 24)	Kang et al. [122]
Liraglutide Effect and Action in Diabetes: Evaluation of Cardiovascular Outcome Results (LEADER^®^)	- Randomized, double-blind, phase 3 study - 9341 participants Age > 50 years DM (non on GLP-1a or DPP-4i) Cardiovascular risk Intervention: once-daily sc 1.8 mg liraglutide or placebo	- The prespecified composite renal outcome occurred in fewer patients in the liraglutide group mainly because fewer participants in the intervention group presented new-onset persistent macroalbuminuria	Mann et al. [123]
A Study of Retatrutide (LY3437943) on Renal Function in Participants With Overweight or Obesity and Chronic Kidney Disease With or Without Type 2 Diabetes	Phase 2 double blind study Age ≥ 18 years old BMI ≥ 27 kg/m^2^ CKD With or without T2DM - Treatment with sc retatruitide or placebo - Estimated 120 participants	Still recruiting (last update was posted on 26 September 2024)	NCT05936151
Effects of Renal Impairment on the Pharmacokinetics of the Dual GIP and GLP-1 Receptor Agonist Tirzepatide	- Phase 1 study - Age 18–85 years - BMI 19–40 kg/m^2^ - Treatment with 5 mg of sc tirzepatide - 45 participants	- No significant relationship between tirzepatide exposure and eGFR. Tirzepatide at a dose of 5 mg is safe in CKD	Urva et al. [124]
Comparison of the Efficacy and Safety of SGLT2i and GLP-1 Receptor Agonists in Obese Patients With Kidney Disease	Age 18–75 years old BMI > 27 kg/m^2^ or WC ≥ 85 cm (♂) ≥ 80 cm (♀) or WHR ≥ 0.9 (♂) ≥ 0.85 (♀) Kidney biopsy confirming ORG On optimized therapy with ACEI/ARBs and/or MRAs for at least 3 months: - Estimated 48 participants - Randomization 1:1:1:1 (RAS blockers: RAS b+SGLT2i: RAS b+GLP-1^α^: RAS b+SGLT2i+GLP-1^α^)	- Still recruiting (last update: 3 April 2024)	NCT06344247
Effects of the SGLT2 inhibitor dapagliflozin on proteinuria in non-diabetic patients with chronic kidney disease (DIAMOND): a randomised, double-blind, crossover trial	- Randomised, double-blind study - Phase 2 study - 53 participants Age 18–75 years Albuminuria 500–3500 mg/g in a 24 h urine collection On a stable dose of RAS blockers for at least one month No DM - Intervention: dapagliflozin 10 mg or placebo for 6 weeks	- No effect of dapaglifozin on proteinuria - Weight reduction - Acute and reversible decline in mGFR	Cherney et al. [125]
Effect of Dapagliflozin on Clinical Outcomes in Patients With Chronic Kidney Disease, With and Without Cardiovascular Disease (Dapa-CKD)	- International, double-blind phase 3 study - 4304 participants: Aged ≥ 18 years eGFR 25–75 mL/min/1.73 m^2^ UACR ≥ 200 and ≤5000 mg/g at visit 1 On RAS blockers - Intervention: dapagliflozin 10 mg once daily or placebo	- Reduced cardiovascular risk in patients with CKD	Wheeler et al. [106]
Beneficial long-term effect of aldosterone antagonist added to a traditional blockade of the renin–angiotensin–aldosterone system among patients with obesity and proteinuria	Prospective cohort study 71 participants Proteinuria > 1 g/24 h On maximum tolerated dosages of ACEIs, ARΒs, or their combination for >6 months Obese and non-obese patients - Intervention: addition of 25 mg/day spironolactone to the baseline therapy of all patients	- Aldosterone antagonist treatment leads to a significant and sustained reduction in proteinuria at 12 months in approximately 65% of obese patients with proteinuric nephropathies, comparable to the efficacy observed in non-obese patients	Morales et al. [102]
Effect of Finerenone on Chronic Kidney Disease Outcomes in Type 2 Diabetes (FIDELIO-DKD: Efficacy and Safety of Finerenone in Subjects With Type 2 Diabetes Mellitus and Diabetic Kidney Disease)	- Double-blind trial - 5734 participants Age ≥ 18 years with T2DM UACR 30–300 mg/g and eGFR 25–60 mL/min/1.73 m^2^ and diabetic retinopathy UACR 300–5000 mg/g and eGFR 25–75 mL/min/1.73 m^2^ On optimized treatment with RAS blockers - Intervention: 10 mg oral finerenone or placebo	- Finerenone resulted in lower risks of CKD progression and cardiovascular events than placebo.	Bakris et al. [126]
Effect of Finerenone on Albuminuria in Patients With Diabetic Nephropathy: A Randomized Clinical Trial	- Randomized, double-blindstudy - 821 participants DM UACR ≥ 30 mg/g eGFR ≥ 30 mL/min/1.73 m^2^ Already on RAS blockers - Intervention: oral, once-daily finerenone in various doses or placebo for 90 days	- Significant dose-dependent decrease in UACR compared to placebo	Bakris et al. [127]

**Table 3:** Comparison of key differences between obesity-related chronic kidney disease and other causes of chronic kidney disease. Abbreviations—ACE: Angiotensin-Converting Enzyme; ARΒs: Angiotensin Receptor Blockers; CKD: Chronic Kidney Disease; eGFR: Estimated Glomerular Filtration Rate; GLP-1 agonists: Glucagon-likepeptide-1 agonists; MRA: Mineralocorticoid-Receptor Antagonist; ORG: Obesity-Related Glomerulopathy; PPARa: Peroxisome Proliferator-Activated Receptor agonists; RAAS: Renin-Angiotensin-Aldosterone-System; RRT: Renal Replacement Therapy; SGLT2 inhibitors: Sodium–Glucose Transport Protein 2 inhibitors; SREBP: Sterol-Regulatory-Element-Binding Protein; T2DM: Type 2 Diabetes Mellitus.

Characteristic	Obesity-Related CKD	Other Causes of CKD
Incidence	Increasing in parallel to the incidence of obesity [14] 15–30% of patients with CKD [16]	- Increasing - Prevalence: 10% to 14% of the general population [130]
Age at presentation	Most common in middle-aged adults (40–65 y) but may be present in children or older adults	Mainly middle-aged (40–65 y) or older adults (>65 y)
Clinical Presentation	Detection of proteinuria, along with normal urinary sediment in obese individuals [14]	Elevated serum creatinine, reduced eGFR
Clinical course	- Stable or slowly progressive subnephrotic proteinuria - However, up to one third of patients with ORG may develop proteinuria in the nephrotic range [14]	- Progressive proteinuria - Nephrotic syndrome - Kidney failure
Serum albumin levels	Normal in most cases [15]	Usually low
Histology	- Oligonephronia - Glomerulomegaly - FSGS, particularly the perihilar variant - Intracellular lipid vacuoles - Less extensive foot process effacement - Focal “diabetoid” changes [14,131]	Depending on the aetiology of CKD: - Glomerular sclerosis - Interstitial infiltration - Interstitial fibrosis - Tubular atrophy
Imaging	Glomerulomegaly (the hallmark of ORG,100% of cases) [131]	- Atrophic kidneys (maximum renal length decreases contemporarily to GFR [132]) - Glomerulomegaly in diabetic kidney disease
Pathophysiology	- Haemodynamic changes (glomerulal hyperfiltration, activation of RAAS) - Lipid accumulation - Insulin resistance - Inflammation and oxidative stress	- Atherosclerotic lesions - Hypoperfusion - Infiltration of kidneys with extrinsic inflammatory cells - Activation and proliferation of extracellular matrix producing cells including myofibroblasts and fibroblasts [130]
Therapeutic approaches	- Weight reduction (hypocaloric diet, bariatric surgery) - ACE inhibitors and ARBs - MRAs - SGLT2 inhibitors, GLP-1 agonists, and other antidiabetic agents - Melatonin - SREBP antagonists, agonists of PPARα, and other agents against lipid accumulation [15] - Combining therapeutic approaches	- ACE inhibitors and ARBs - SGLT2 inhibitors - Avoidance of nephrotoxic-agent RRT - Combining therapeutic approaches

## Data Availability

Not applicable.

## Abbreviations

ACE: Angiotensin-Converting Enzyme; ACEI: Angiotensin-Converting Enzyme Inhibitor; ARBs: Angiotensin Receptor Blockers; BMI: Body Mass Index; BSA: Body Surface Area; CKD: Chronic Kidney Disease; CT: Computed Tomography; DDP-4 inhibitors: Dipeptidyl peptidase 4 inhibitors; DKD: Diabetic Kidney Disease; DM: Diabetes Mellitus; eGFR: estimated Glomerular Filtration Rate; ER: Endoplasmic Reticulum; EXE: exenatide; FFAs: Free Fatty Acids; FSGS: Focal Segmental Glomerulosclerosis; Gal-3: Galectin-3; GFR: Glomerular Filtration Rate; GIP: Glucose-dependent Insulinotropic Peptide; GH: Glomerular Hyperfiltration; GLP-1: Glucagon Like Peptide-1; KIM-1: Kidney Injury Molecule-1; miRNAs: MicroRNAs; nsMRAs: non-steroidal Mineralocorticoid Receptor Antagonists; MRI: Magnetic Resonance Imaging; NGAL: Neutrophil-Gelatinase-Associated Lipocalin; NGS: Next-Generation Sequencing; ORG: Obesity-Related Glomerulopathy; PCR: Polymerase Chain Reaction; PPAR: Peroxisome-proliferator-activated receptor; PUFT: Pararenal and perirenal ultrasonographic fat thickness; RAS b: Renin Angiotensin System blocker; RAAS: Renin Angiotensin Aldosterone System; ROS: Reactive Oxygen Species; RRT: Renal Replacement Therapy; SCr: Serum Creatinine; sCysC: Cystatin C; SGLT-2: Sodium Glucose Cotransporter-2; SGLT2i: Sodium–Glucose Transport Protein 2 inhibitor; SREBP-2: Sterol-Regulatory-Element-binding protein 2; T2DM: Type 2 Diabetes Mellitus; TGF-β: Transforming Growth Factor β;TNF-a: Tumor Necrosis Factor alpha; UACR: Urine Albumine–Creatinine Ratio; WC: Waist Circumference; WHR: Waist-to-Hip-Ratio; WHO: World Health Organization.
